# *Salmonella enterica* serovar Choleraesuis vector delivering a dual-antigen expression cassette provides mouse cross-protection against *Streptococcus suis* serotypes 2, 7, 9, and 1/2

**DOI:** 10.1186/s13567-022-01062-9

**Published:** 2022-06-22

**Authors:** Yu-an Li, Yanni Sun, Yang Fu, Yuqin Zhang, Quan Li, Shifeng Wang, Huoying Shi

**Affiliations:** 1 College of Veterinary Medicine, Yangzhou University, Yangzhou, 225009 Jiangsu China; 2 Jiangsu Co-Innovation Center for the Prevention and Control of Important Animal Infectious Diseases and Zoonoses, Yangzhou, China; 3 Department of Infectious Diseases and Immunology, College of Veterinary Medicine, University of Florida, Gainesville, FL 32611-0880 USA; 4 Joint International Research Laboratory of Agriculture and Agri-Product Safety, Yangzhou University (JIRLAAPS), Yangzhou, China

**Keywords:** *Salmonella* Choleraesuis vector, *Streptococcus suis*, dual antigen expression cassettes, universal vaccine

## Abstract

A universal vaccine protecting against multiple serotypes of *Streptococcus suis* is urgently needed to improve animal welfare and reduce the consumption of antibiotics. In this study, a dual antigen expression cassette consisting of SS2-SaoA and SS9-Eno was delivered by a recombinant *Salmonella* Choleraesuis vector to form the vaccine candidate rSC0016(pS-SE). SaoA and Eno were simultaneously synthesized in rSC0016(pS-SE) without affecting the colonization of the recombinant vector in the lymphatic system. In addition, the antiserum of mice immunized with rSC0016(pS-SE) produced a broader and potent opsonophagocytic response against multiple serotypes of *S. suis*. Finally, rSC0016(pS-SE) provided mice with a 100% protection against a lethal dose of parent *S. suis* serotype 2 and serotype 9, and provided 90% and 80% protection against heterologous *S. suis* serotype 7 or 1/2. These values were significantly higher than those obtained with rSC0016(pS-SaoA) or rSC0016(pS-Eno). Together, this study serves as a foundation for developing a universal vaccine against multiple serotypes of *S. suis*.

## Introduction

*Streptococcus suis* is an important zoonotic pathogen, which causes substantial economic losses in the pig industry worldwide and many infections in humans [1, 2]. There are 35 serotypes of *S. suis*; serotypes 1/2, 2, 3, 7, and 9 (SS1/2, SS2, SS3, SS7, and SS9) are by far the most prevalent worldwide [2]. Vaccination is beneficial in reducing antibiotics consumption and slowing down antibiotic resistance development [3]. However, there are bottlenecks in the development of a *S. suis* vaccine. The expanded culture of *S. suis* requires a financially costly medium, which may render difficult the development of inactivated vaccines in less developed regions [3]. In addition, some vaccine candidates fail to induce opsonically active antibodies and thus fail to provide adequate protection [4–6]. Most importantly, existing vaccines lack cross-reactivity to ensure the protection against heterologous strains with multiple serotypes [7–10]. Therefore, developing an economical and effective universal vaccine is necessary to prevent disease with *S. suis* [1, 3].

An ideal *S. suis* vaccine should induce cross-protection against multiple serotypes. Among the immunogenic proteins tested as *S. suis* vaccine candidates, only Sao [11, 12], Eno [13], and PrsA [14] have been reported for their capacity to induce cross-protection. Conservative antigens among multiple serotypes are especially useful in veterinary practice if they protect against challenges by strains of heterologous serotypes. Sao is a highly conserved antigen and provides cross-protection against *S. suis* serotypes 1, 2, and 7 [6, 11, 12]. In addition, Sao protected pigs against aerosol-challenge with *S. suis* and induced opsonophagocytic activity (OPA) antibody against *S. suis* [6]. OPA antibody has been shown to be closely associated with protective immune responses against S. suis [6, 11, 15]. Another protein, Enolase (Eno), a 52-kDa surface fibronectin-binding protein [16], has also been shown to provide protection against *S. suis* serotypes 2 and 7 in a mouse model when mixed with Freund’s Complete Adjuvant (FCA) [13]. These studies have shown that Sao and Eno have high immunogenicity and cross-reactivity. Both are potent candidates as *S. suis* universal vaccine.

Vaccines containing multiple antigens confer better protection than those containing a single antigen [17, 18].

A vaccine containing both muramidase-released protein (MRP) and extracellular factor (EF) protects pigs against challenge with *S. suis* serotype 2 virulent strain, while vaccines containing either MRP or EF alone were not protective [19]. Antigen combinations from different serotypes of BTV-*Bluetongue* virus not only provide protection against the parental serotype but also provide partial cross-protection against heterologous serotypes [20]. The combination of multiple antigens may bring about a synergistic effect. The multicomponent vaccine 5CVMB, which contains five serogroup B *meningococcus* (MenB) antigens, formulated with aluminum hydroxide induced strong immune responses. The bactericidal antibodies induced by 5CVMB were more potent than those induced by the individual antigen. The novel 5CVMB vaccine expands the vaccine coverage and avoids the selection of escape mutants [21].

Attenuated *Salmonella* vector as an antigen presentation platform can induce superior mucosal antibody response, which is critical against mucosal pathogens [22, 23]. In addition, the *Salmonella* vector can colonize the host lymphatic system, thereby continuously stimulating immune cells and ultimately inducing a long-term immune response [22–24]. Most importantly, attenuated *Salmonella* has known adjuvant properties that can enhance the humoral and cellular immune responses induced by foreign antigens, making it an excellent vector for presenting heterologous antigens [25, 26]. In our previous study, the attenuated *S.* Choleraesuis vector delivering SaoA from *S. suis* serotype 2 provided heterologous protection against SS7 in mice or piglets. However, it still could not induce protection against heterologous serotypes [12]. In this study, a dual expression cassette plasmid containing SS2-SaoA and SS9-Eno (pS-SE) was introduced into *S*. Choleraesuis rSC0016 to form the recombinant *S*. Choleraesuis vector rSC0016(pS-SE). The immunogenicity and the cross-protection efficiency of rSC0016(pS-SE) against multiple heterologous *S. suis* were evaluated in mice.

## Materials and methods

### Ethical statement

All animal experiments were authorized by the Department of Science and Technology of Jiangsu Province with a license number of SCXK(SU) 2018-0009. All experimental procedures were approved by the Jiangsu Laboratory Animal Welfare and Ethics guidelines of the Jiangsu Administrative Committee of Laboratory Animals to minimize animal pain.

### Bacterial strains, plasmids, and culture conditions

Bacterial strains and plasmids utilized in this study are described in Table 1. *S. suis* serotype 7 (SS7) virulent strain SH04805, *S. Suis* serotype 9 (SS9) virulent strain GZ0565, and *S. Suis* serotype 1/2 (SS1/2) virulent strain 2651 were kindly provided by Professor Huochun Yao (Nanjing Agricultural University). *S. suis* serotype 2 (SS2, CVCC3928) was purchased from China Veterinary Culture Collection Center. Plasmid pYA3493 is an Asd^+^ vector with a P_trc_ promoter. Plasmid pS-SE, derived from pYA3493, carries a dual antigen expression cassette consisting of SS2-SaoA and SS9-Eno. Plasmid pS-Eno, derived from pYA3493, carries an SS9-Eno. Plasmid pS-SaoA was described in our previous study [12]. *S*. Choleraesuis strains were prepared as previously described [12, 27]. The live attenuated *S*. Choleraesuis strain rSC0016 delivering plasmid pYA3493, pS-SaoA, pS-Eno, or pS-SE were grown in LB broth with both 0.2% L-arabinose and 0.2% D-mannose. LB broth and tetrathionate medium (BD Difco) were used for the enrichment of *S.* Choleraesuis from mouse tissues. Nutrient Broth (NB) and MacConkey agar (Difco) were used for phenotype characterization. When required, media were supplemented with 2,6-diaminopimelic acid (DAP; 50 µg/mL), L-arabinose (0.2% wt/vol), D-mannose (0.2% wt/vol) or sucrose (5% wt/vol). Bacterial growth was monitored with a spectrophotometer at OD_600_ and by direct plating for colony counts.

**Table 1:** **Strains, plasmids and primers**.

Strain or plasmid or primer	Characteristics^a^ or sequences^b^	Source, reference or function
Bacterial strains		
BL21	For expression the recombinant plasmids	Invitrogen
χ7213	*thi-1 thr-1 leuB6 fhuA21 lacY1 glnV44 asdA4 recA1 RP4 2-Tc::Mu pir;* Km^r^	[48]
rSC0016	ΔP_crp527_::TT *araC*P_BAD_*crp* Δ*pmi-2426* Δ*relA199::araC*P_BAD_*lacI*TT Δ*sopB1686* Δ*asdA33*	[29]
* Streptococcus suis* serotype 2	Wild type, virulent, CVCC3928	Lab stock
* Streptococcus suis* serotype 7	Wild type, virulent, SH04805	Provided by Professor Huochun Yao
* Streptococcus suis* serotype 9	Wild type, virulent, GZ0565	Provided by Professor Huochun Yao
* Streptococcus suis* serotype 1/2	Wild type, virulent, 2651	Provided by Professor Huochun Yao
Plasmids		
pYA3493	Asd^+^; pBR *ori*, P_trc_ promoter, β-lactamase signal sequence-based periplasmic secretion plasmid	[49]
pS-SaoA	pYA3493 with SaoA, P_trc_ promoter	[29]
pS-Eno	pYA3493 with Eno, P_trc_ promoter	This study
pS-SE	pYA3493 with a dual antigen expression cassettes consisting of SS2-SaoA and SS9-Eno, P_trc_ promoter	This study
pET28a-SaoA	SS2-SaoA expression vector, T7 promoter; Km^r^	[29]
pET28a-Eno	SS9-Eno expression vector, T7 promoter; Km^r^	This study
Primers		
SS9-Eno-F	CG**CTGCAG**AGGACGCAAAAAATGAAAAAGACGGCTATCGC	The ORF of SS9-Eno
SS9-Eno-R	GC**AAGCTT**TTACTTTTTCAAGTTATAGAAT	
SE-F	AT**CCCGGG**CAACCTGATGGGGGCCAGG	The ORF of SaoA-Eno
SE-R	GC**AAGCTT**TTACTTTTTCAAGTTATAGAAT	

^*a*^*Km*^*r*^ Kanamycin resistance. ^b^Underlined nucleotides denote enzyme restriction sites.

### Construction and plasmid stability of the attenuated *S*. Choleraesuis vector with plasmids pYA3493, pS-Eno and pS-SE

*S*. Choleraesuis vector, rSC0016, was described in a previously study [12]. The codon-optimized SS9-*eno* was synthesized by TaKaRa Bio and cloned into pYA3493 with *Pst* Ι and *Hind* III to generate pS-Eno. An OmpA signal peptide was fused at the amino-terminal of Eno in pS-Eno to replace the original Bla/ss signal peptide. Similarly, the codon-optimized SE operon fusion of SS2-*saoA* and SS9-*eno* was synthesized by TaKaRa Bio and cloned into pYA3493 with *Sma* Ι and *Hind* III to generate pS-SE. Bla/ss or OmpA signal peptides were fused at the amino terminal of SaoA or Eno in pS-SE, respectively. Subsequently, Asd + plasmids pS-Eno and pS-SE were transformed into a live attenuated *S*. Choleraesuis vaccine strain, rSC0016, to generate rSC0016(pS-Eno) and rSC0016(pS-SE). To investigate the stabilities of recombinant plasmid pS-Eno and pS-SE in rSC0016, the strains harboring pS-Eno or pS-SE were maintained in an LB medium with 1:500 dilutions for 50 generations [27]. Plasmids pS-Eno and pS-SE were extracted at the 50th generation and confirmed with double enzymatic digestion. PCR verified mutations of the attenuated strain rSC0016 with correspondent primers. Production of Eno in rSC0016(pS-Eno) or SaoA and Eno in rSC0016 (pS-SE) were finally tested by western blot with corresponding antiserum [12, 28].

### Western blot analysis of LacI, SaoA, and Eno expression

To evaluate arabinose dependent regulation of SaoA and Eno, rSC0016(pS-SE) were grown in NB medium with 0.2% L-arabinose (Sigma) at 37 °C. When the culture reached an OD_600_ of 0.8, it was diluted 1:100 into fresh NB without L-arabinose and grown to an OD_600_ of 0.8. This process was repeated 5 times. One mL of culture was collected from each passage and prepared for western blot analysis [12]. Briefly, the total protein samples were normalized and separated on a 10% (wt/vol) SDS-PAGE gel and transferred onto nitrocellulose membranes. Western blot analysis was performed using a rabbit anti-LacI, anti-SaoA and anti-Eno antisera, followed by HRP conjugated goat anti-rabbit polyclonal antibody (Sigma) [12, 28]. Densitometry was quantified using Image J2 software [29].

### *S*. Choleraesuis subcellular fractionation

To evaluate the subcellular localization of synthesized SaoA and Eno in rSC0016(pS-SaoA), rSC0016(pS-Eno), and rSC0016(pS-SE), mid-exponential growth phase cultures of the corresponding strains were harvested by centrifugation at 4 °C. Periplasmic fractions were prepared by modifying the lysozyme-osmotic shock method as previously described [30]. Briefly, cultures were grown in NB medium to an OD_600_ of 0.8 and induced with 0.5 mM IPTG (isopropyl-d-thiogalactopyranoside) for 3 h. The bacteria density was normalized by absorbance at OD_600_ to ensure that the samples in each lane contained the same number of bacteria. One mL of culture was collected for western blot analysis. The supernatant fluid was filtered and saved for the analysis of secreted proteins. Equal volumes of periplasmic, cytoplasmic, and supernatant fractions and total protein lysate samples were separated on a 10% SDS-PAGE gel and transferred onto a nitrocellulose membrane for western blot analysis. Densitometry was quantified using Image J2 software [29].

### Preparation of SaoA and Eno proteins

To obtain recombinant Eno from *E. coli*, the SS9-*eno* gene was optimized for *E. coli*-preferred codon and synthesized by TaKaRa Bio. SS9-*eno* gene was inserted into the pET-28a vector using *Pst* I and *Hind* III restriction enzymes. The pET-28a-Eno plasmid was transformed into *E. coli* strain BL21(DE3) for expression.

His-tagged SaoA or Eno fusion proteins were prepared as previously described [28]. Briefly, *Escherichia coli* strain BL21 carrying pET28a-SaoA or pET28a-Eno were grown to mid-log phase (OD_600_ values of 0.6–0.9) in LB medium with kanamycin at 30 °C and induced with 0.3 mM IPTG for 8 h. The recombinant SaoA or Eno proteins were purified by Ni-chelating affinity gel (Sigma) according to the instruction manual.

### Distribution of *S.* Choleraesuis vector strains in BALB/c mice

The *S.* Choleraesuis strains, rSC0016(pYA3493), rSC0016(pS-SaoA), rSC0016(pS-Eno) and rSC0016(pS-SE), were grown statically overnight in LB broth supplemented with 0.2% L-arabinose and D-mannose at 37 °C. A 500 μL of overnight culture was inoculated into 50 mL of LB broth containing the appropriate supplements and grown with aeration to an OD_600_ of 0.85 to 0.9 at 37 °C. Cells were pelleted by centrifugation at room temperature (8000 rpm for 10 min). The pellets were resuspended in 300 μL of PBS. The colonization assay for recombination *S.* Choleraesuis vector strains was performed as described previously [27]. Eighty mice were randomly divided into 4 groups with 20 mice in each group. Groups of mice were orally inoculated with 1 ± 0.2 × 10^9^ CFU of *S.* Choleraesuis strains of rSC0016(pYA3493), rSC0016(pS-SaoA), rSC0016(pS-Eno) or rSC0016(pS-SE), respectively. Five mice in each group were euthanized at each of the time points (days 3, 7, 14, and 21 post-inoculated). Subsequently, Peyer’s patches, spleen, and liver of each mouse were collected and homogenized in PBS after weighing. The CFUs of bacteria were determined using dilution plating on MacConkey agar plates containing 0.2% L-arabinose (wt/vol), 0.2% D-mannose (wt/vol) and 1% D-lactose (wt/vol). The residual 900 mL of homogenized tissues were inoculated into 5 mL tetrathionate medium for *Salmonella* enrichment when no colonies were observed on the MacConkey agar plates. Samples that were negative by direct plating and positive by enrichment were recorded as 10 CFU/g, while those negative were recorded as 0 CFU/g. The assay was performed twice, and the data was similar and pooled for analysis.

### Mouse immunization

Six-week-old female BALB/c mice were deprived of food and water for 4 h before oral administration of *S*. Choleraesuis strains. Food and water were returned to the animals 30 min later. To determine bacterial titers, dilutions of *S*. Choleraesuis strains were plated onto LB agar with 0.2% L-arabinose. Two hundred and fifty mice were randomly divided into 5 groups, with 50 mice per group. Groups of mice were orally inoculated with 1 ± 0.2 × 10^9^ CFU of *S*. Choleraesuis strains of rSC0016(pYA3493), rSC0016(pS-SaoA), rSC0016(pS-Eno), rSC0016(pS-SE) or an equal volume of PBS, respectively. Mice were boosted with the same dose of the same strain after three weeks. Blood and vaginal secretion specimens from each mouse in a random subset (10 mice) in each group were collected 3 weeks after the first immunization and 2 weeks after boosting. Serum fractions were stored at −20 °C. Vaginal secretion specimens were collected by washing with 50 μL of PBS before storage at −20 °C.

### Enzyme-linked immunosorbent assay (ELISA)

The procedures used for antibodies measurement were described previously [12, 27]. Briefly, purified SaoA (50 ng per well) or Eno (50 ng per well) in sodium carbonate-sodium bicarbonate buffer (pH9.6) were coated in Nunc-Immuno MaxiSop 96-well plates (Corning, NY, USA). Plates were incubated overnight at 4 °C. Free binding sites were blocked with 0.5% BSA. A 100 μL volume of diluted serum was added to individual wells in duplicate and incubated for 3 h at room temperature. Plates were treated with Peroxidase-conjugated goat anti-mouse IgG, IgG1, or IgG2a (Abcam) for sera and IgA (BD) for vaginal secretions.

### Opsonophagocytic assay

The OPA was performed as previously described [31]. Briefly, a random subset of 10 mice was selected from each group on the 14th day after the booster. Subsequently, serum from each mouse of each subset was collected and twofold serially diluted for opsonic killing assay. Porcine polymorphonuclear leukocytes (PMNs) were separated by Ficoll-Hypaque (Haoyang Biological Manufacture Co. Ltd., Tian-jin, China). Cell viability was evaluated higher than 90% by Trypan blue exclusion. Log-phase SS2, SS7, SS9, and SS1/2 were opsonized with 50 μL of the diluted serum samples at 37 °C for 15 min, respectively. PMNs at a concentration of 1 × 10^7^ cells/mL were mixed with equal volume (100 μL) of 1 × 10^7^ CFU/mL opsonized bacteria. After incubation with PMNs for 1 h at 37 °C, serial dilutions of assay samples were plated onto THB agar and then cultured overnight at 37 °C. Bacteria were enumerated the following day. The serum dilution that leaded to killing of 50% of the assay bacteria was defined as the OPA titer. The mean of three independent CFU counts for each sample was used to calculate the survival ratio.

### Mouse challenge with *S. suis*

On the 14^th^ day after the booster, 10 mice in each group were challenged intraperitoneally with 10 × LD_50_ of SS2, SS7, SS9 and SS1/2, respectively. Mouse survival and health status were observed and recorded daily for 14 days. Tissue samples from the brain and lung were fixed in 4% paraformaldehyde. After paraffin embedding, tissue sections were cut and stained with hematoxylin and eosin (H&E). The histological sections of each mouse were scored pathologically as follows. For lung, 0: normal; 1: congestion; 2: interstitial thickening; 3: inflammatory cell infiltration in bronchial submucosa; 4: a mass of inflammatory cell infiltration in bronchial submucosa. For brain, 0: normal; 1: congestion; 2: a few inflammatory cells infiltrate the meninges; 3: a mass of inflammatory cells meninges; 4: meningeal hemorrhage with extensive inflammatory cell infiltration. This experiment was performed twice, with each group (50 mice) receiving approximately the same dose of vaccine. The results from both experiments were similar, and the data were pooled for analysis.

### Statistical analysis

Statistical analyses on ELISA were presented as geometric means and standard deviations for all assays. A Mann–Whitney U Test (GraphPad Software, Inc.) was applied to compare the distribution of the *S*. Choleraesuis in tissues of mice and densitometric analysis. The log-rank (Mantel–Cox) test was used to compare the survival after the challenge. For all tests, a *P* < 0.05 was considered statistically significant.

## Results

### Construction of a dual expression cassette vector

**Figure 1:**
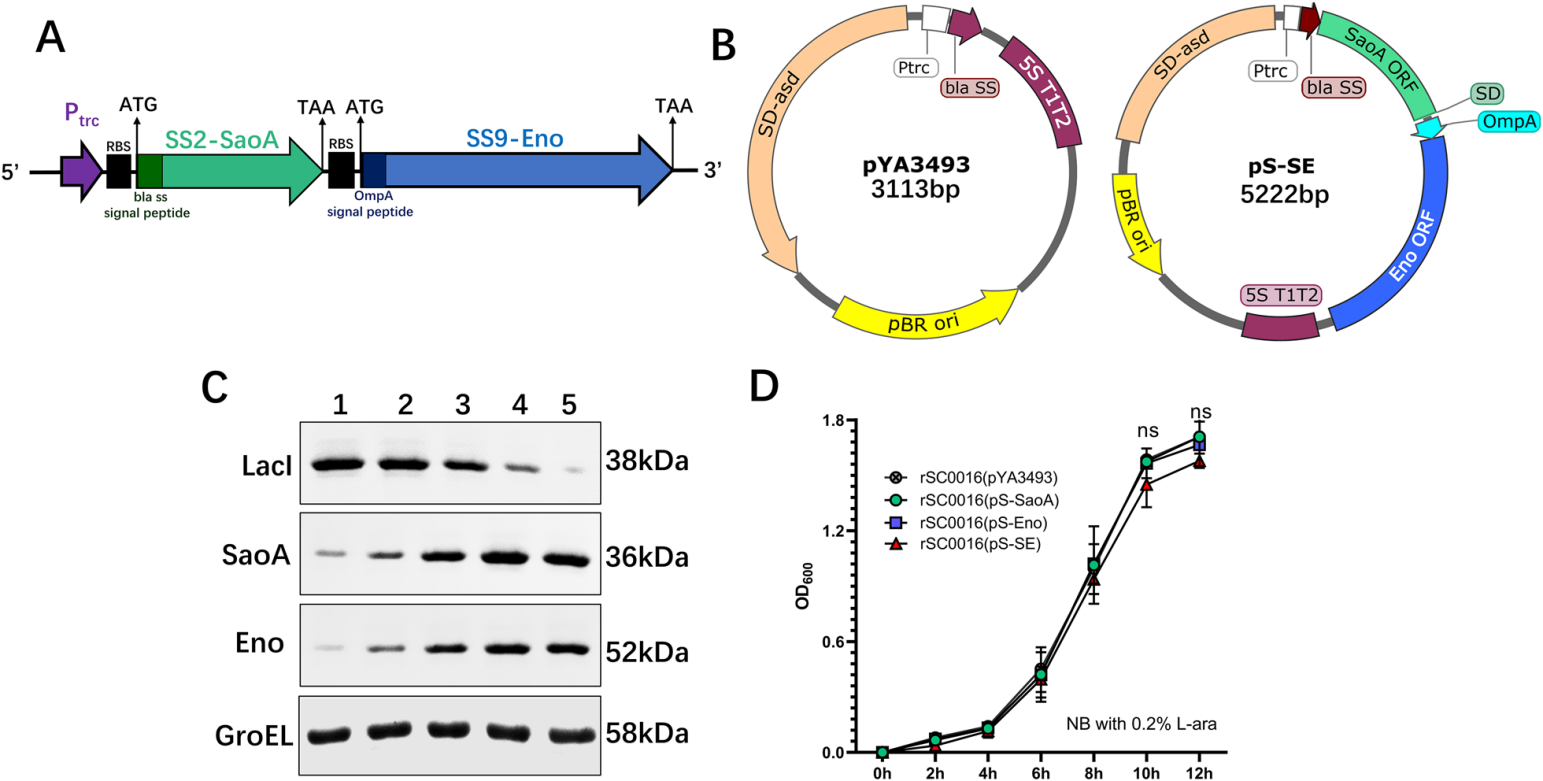
**Design of a dual expression cassette for SaoA and Eno and phenotypic identification. A** Schematic diagram of operon fusion of *saoA* and *eno* dual expression cassette. A ribosomal binding site was added between the open reading frames of SaoA and Eno, and the nucleotide sequence encoding OmpA secretion signal peptide was fused with the open reading frame of Eno. Both *saoA* and *eno* are regulated by the P_trc_ promoter. **B** Plasmid maps of control vector plasmid pYA3493 and pS-SE (SaoA-Eno). **C** Regulated decreased synthesis of LacI and regulated delayed synthesis of SaoA or Eno proteins in rSC0016(pS-SE) containing Δ*relA*::*araC* P_BAD_ *lacI* TT mutation. Strain rSC0016(pS-SE) was grown in NB with L-arabinose and D-mannose (Lane 1) and then diluted 1:10 into fresh NB without L-arabinose and D-mannose until OD_600_ to 0.8. The process was repeated four times (Lane 2–5). Each lane was loaded with around 2.5 × 10^7^ CFU bacteria. Synthesis of LacI, SaoA, Eno and GroEL were detected by western blot using correspondent antibodies. **D** Growth of rSC0016(pYA3493), rSC0016(pS-SaoA), rSC0016(pS-Eno), and rSC0016(pS-SE) in Nutrient broth with 0.2% L-arabinose and no 0.2% D-mannose. (ns) means that there is no significant difference between rSC0016(pS-SE) and rSC0016(pS-SaoA) or rSC0016(pS-Eno).

SaoA protein homology among SS2, SS9, and SS1/2 serotypes is over 94.2%. Eno protein homology among these strains is over 99.8%. In addition, there was the same one amino acid difference between SS9-Eno and SS2-Eno, SS7-Eno, and SS1/2-Eno at position 250 (SS9-Eno: Phenylalanine; SS2-Eno, SS7-Eno, and SS1/2-Eno: Tyrosine). To express SS2-*saoA* and SS9-*eno* on the same vector without interference, operon fusion of *saoA* and *eno* was separated by a ribosome binding sites (RBS) to form two separate expression cassettes under the regulation of the P_trc_ promoter (Figure 1A). Each gene has its own secretion signal. The dual expression cassette fragment was digested with *Sma* Ι and *Hind* III and cloned into the prokaryotic expression vector pYA3493 to form the expression plasmid pS-SE (SE represents for SaoA-Eno; Figure 1B).

### Regularly delayed synthesis of SaoA and Eno in rSC0016(pS-SE)

Strain rSC0016 carries a Δ*relA199*::*araC* P_BAD_ *lacI* TT mutation, in which an arabinose-regulated *araC* P_BAD_ promoter controls the production of LacI. LacI inhibits the P_trc_ promoter, which drives the expression of *saoA* and *eno*. The combination of chromosomal *lacI* expression and P_trc_ transcriptional regulation of antigen gene expression comprises the delayed antigen synthesis system. To evaluate the delayed antigen synthesis regulated by LacI, the pS-SE plasmid was introduced into rSC0016. After growing in the NB medium containing 0.2% arabinose, the strain rSC0016(pS-SE) was passaged 4 times in the medium without arabinose. The equal loads of the samples were confirmed by measuring GroEL as an internal reference. As the number of passages increased, arabinose was gradually diluted and the synthesis of LacI decreased, while the synthesis of SaoA or Eno in strain rSC0016(pS-SE) gradually increased (Figure 1C). These results showed that the synthesis of both SaoA and Eno in the dual expression cassette was regulated by the delayed antigen synthesis system.

### Growth characteristics and genetic stability of rSC0016(pS-SE)

The loading of foreign antigen may slow the vector growth, which may decrease the immunogenicity of the vectored antigen [22]. rSC0016(pS-SE) shared similar growth characteristics with rSC0016(pS-SaoA) or rSC0016(pS-Eno), suggesting that the dual expression cassette does not slow down the vector growth (Figure 1D). This result demonstrated that the dual expression cassette did not cause an obvious metabolic burden on the growth of rSC0016. To measure the stability of plasmid pS-SE in rSC0016, rSC0016 containing plasmid pS-SE were cultured for 50 generations. By PCR or endonuclease digestion, it was found that all rSC0016(pS-SE) colonies (200 clones/generation) contained pS-SE plasmids, indicating that pS-SE could be stably maintained in rSC0016 vaccine strains. The cells obtained from the 50th generation culture can synthesize similar amounts of SaoA and Eno to the first generation (data not shown), suggesting that SaoA and Eno are stably synthesized in rSC0016(pS-SE).

### Expression levels of SaoA and Eno in rSC0016(pS-SE)

**Figure 2:**
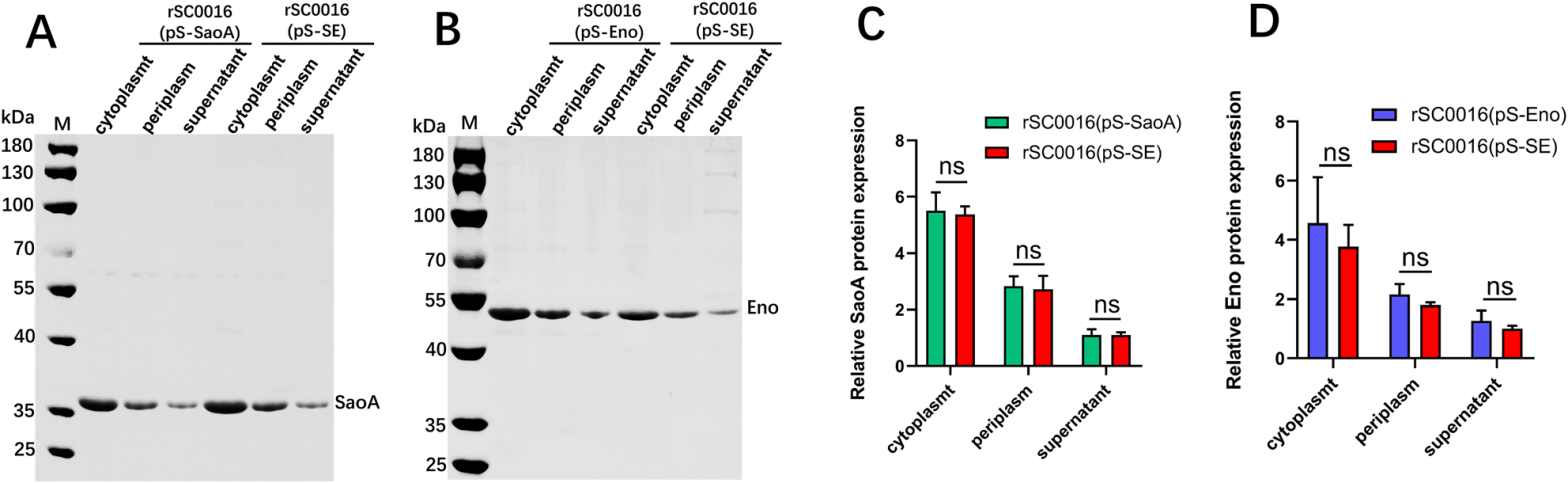
**Expression level and subcellular localization of SaoA or Eno in rSC0016(pS-SE) with the double expression cassette. **Subcellular fractions of SaoA and Eno in rSC0016(pS-SaoA) (**A**), rSC0016(pS-Eno) (**B**) and rSC0016(pS-SE) (**A**, **B**) from cells grown in NB detected by western blot. Representative images in **A** or **B** are from one of the three representative experiments. Densitometry was analyzed with the Image J software (**C**, **D**). ns: not significant.

Transcription and translation efficiency of the open reading frame at the far end of the operon in the dual expression cassette may be reduced [32]. To explore the expression of *eno* at the far end of the operon fusion in pS-SE dual expression cassette, the levels of SaoA or Eno production in the corresponding strains were compared by western blot densitometry. Bands specifically binding to SaoA or Eno antiserum could be detected in the cytoplasm, periplasm, and supernatant of rSC0016(pS-SE) (Figures 2A and B), suggesting that both SaoA and Eno can be synthesized simultaneously in rSC0016 and secreted into the periplasm and extracellularly. In addition, the synthesis of either SaoA (Figure 2C) located at the proximal end of the promoter or Eno (Figure 2D) located at the distal end of the promoter was not significantly different from SaoA or Eno with a single expression cassette.

### Colonization of mouse tissues after oral immunization with rSC0016(pS-SE)

**Figure 3:**
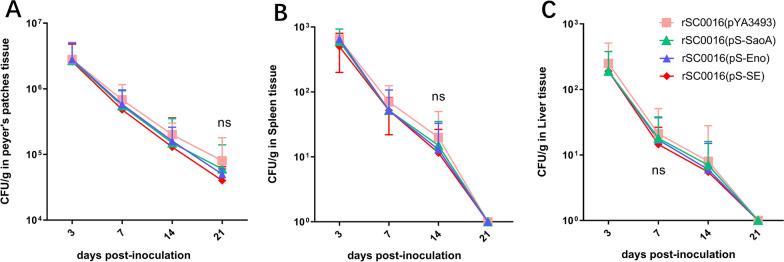
**Distribution of** *S***.** **holeraesuis in BALB/c mice.** Four groups of mice were orally infected with doses of 1 × 10C^9^ CFU of rSC0016(pYA3493), rSC0016(pS-SaoA), rSC0016(pS-Eno) and rSC0016(pS-SE). Bacterial counts were recovered from Peyer’s patches (**A**), spleen (**B**), and liver (**C**) tissues at 3, 7, 14, and 21 days post-infection. Colonies were recorded as CFU/g for organ samples. Data are expressed as the mean ± standard deviations of the five infected mice. (ns) means that there is no significant difference between rSC0016(pS-SE) and rSC0016(pS-SaoA) or rSC0016(pS-Eno) treated groups.

Colonization reflects the interaction between live vector and lymphoid tissues, which is closely related to the immunogenicity of the vector [33, 34]. To evaluate the colonization of rSC0016(pS-SE) in systemic lymphoid tissues, rSC0016 carrying empty vector pYA3493, single expression cassette pS-SaoA, pS-Eno, and dual expression cassette pS-SE was orally given to mice at a dose of 1 × 10^9^ CFU. The bacterial colonization of Peyer’s patches, spleen, and liver were counted at day 3, 7, 14, and 21 after inoculation. There was no significant difference among the colonization levels of rSC0016(pS-SaoA) or rSC0016(pS-Eno) with a single antigen and rSC0016(pS-SE) with dual expression cassette in Peyer’s patches, spleen, and liver (Figure 3). These results indicate that the presentation of dual expression cassettes did not damage the colonization of the recombinant vector in mice.

### Serum IgG and mucosal IgA responses to SaoA or Eno

**Figure 4:**
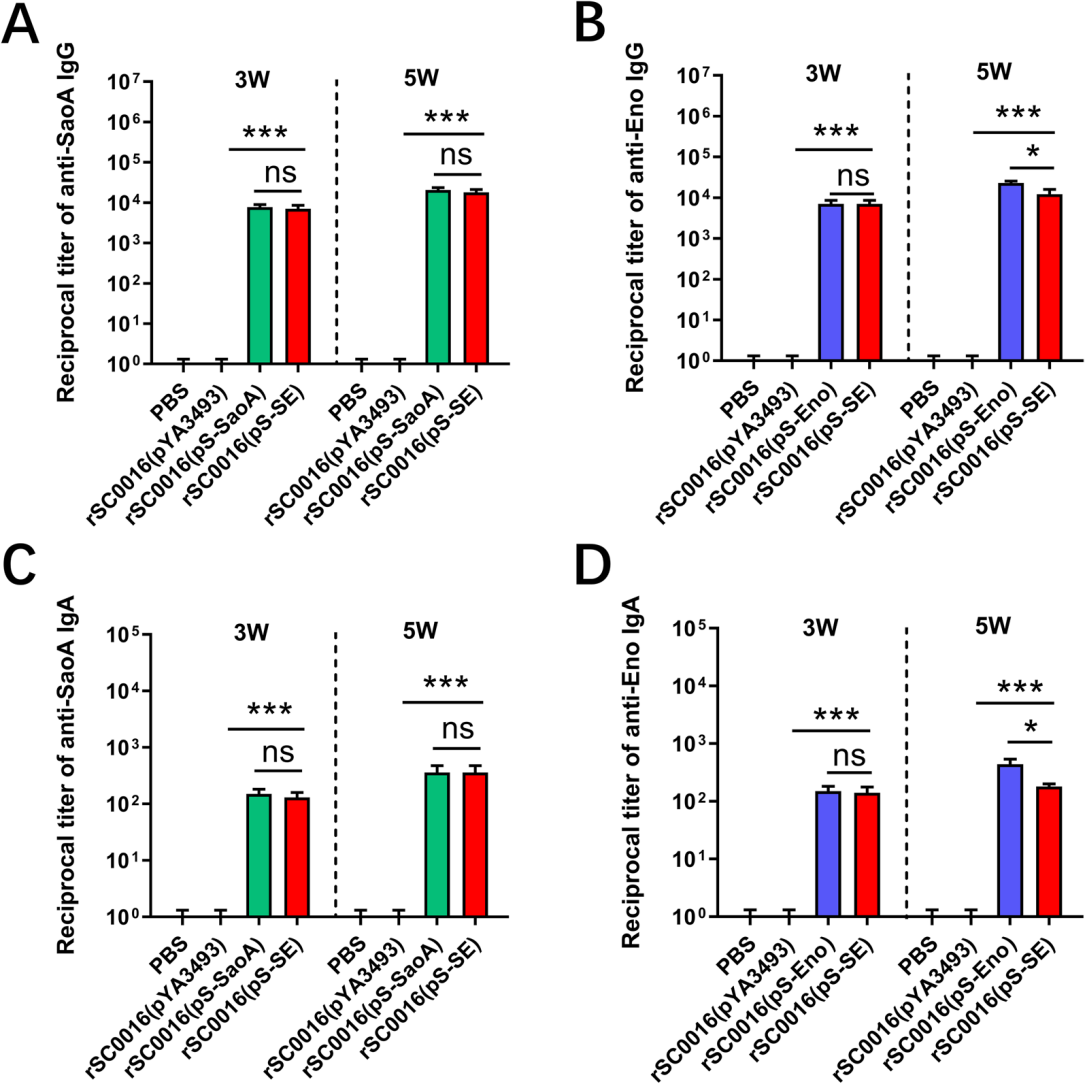
**Antibody responses in mice. **Serum IgG responses to SaoA (**A**) or to Eno (**B**) and vaginal IgA responses to SaoA (**C**) or to Eno (**D**) were measured by ELISA. The data represent reciprocal antibody titers in sera from ten mice orally immunized with attenuated *Salmonella* carrying either pS-SaoA, pS-Eno, pS-SE, or pYA3493 (empty vector) and PBS 3 weeks after the first immunization and 2 weeks after boosting. Serum and vaginal wash obtained from individual mice were serially diluted to obtain titers, starting from either 1:50 or 1:10. Error bars represent variation between mice. *ns* not significant, *P < 0.05; ***P < 0.001. No antibody responses were detected to antigen tested in mice immunized with only PBS or in pre-immune sera from vaccinated mice. ELISA was performed twice with identical results.

To evaluate the humoral and mucosal immune responses induced by rSC0016(pS-SE), serum antibody IgG titers or vaginal mucosal IgA titers of immunized mice were measured 3 weeks after the first immunization and 2 weeks after boosting. Compared with the PBS group or rSC0016(pYA3493) empty vector group, mice orally immunized with rSC0016(pS-SaoA), rSC0016(pS-Eno) and rSC0016(pS-SE) induced higher serum IgG and vaginal IgA titers against SaoA or Eno. Three weeks after the first immunization, the titers of serum IgG and mucosal IgA specific to SaoA or Eno induced by rSC0016(pS-SE) were similar to those of rSC0016(pS-SaoA) or rSC0016(pS-Eno) presenting a single antigen. Two weeks after the booster immunization, there was no significant difference in the specific IgG and IgA titers of SaoA between rSC0016(pS-SE) and rSC0016 (pS-SaoA) (Figures 4A and C). However, the Eno-specific IgG and IgA titers induced by rSC0016(pS-SE) were significantly lower than those induced by rSC0016(pS-Eno) (Figures 4B and D; *P* < 0.05).

### IgG isotype analyses

**Figure 5:**
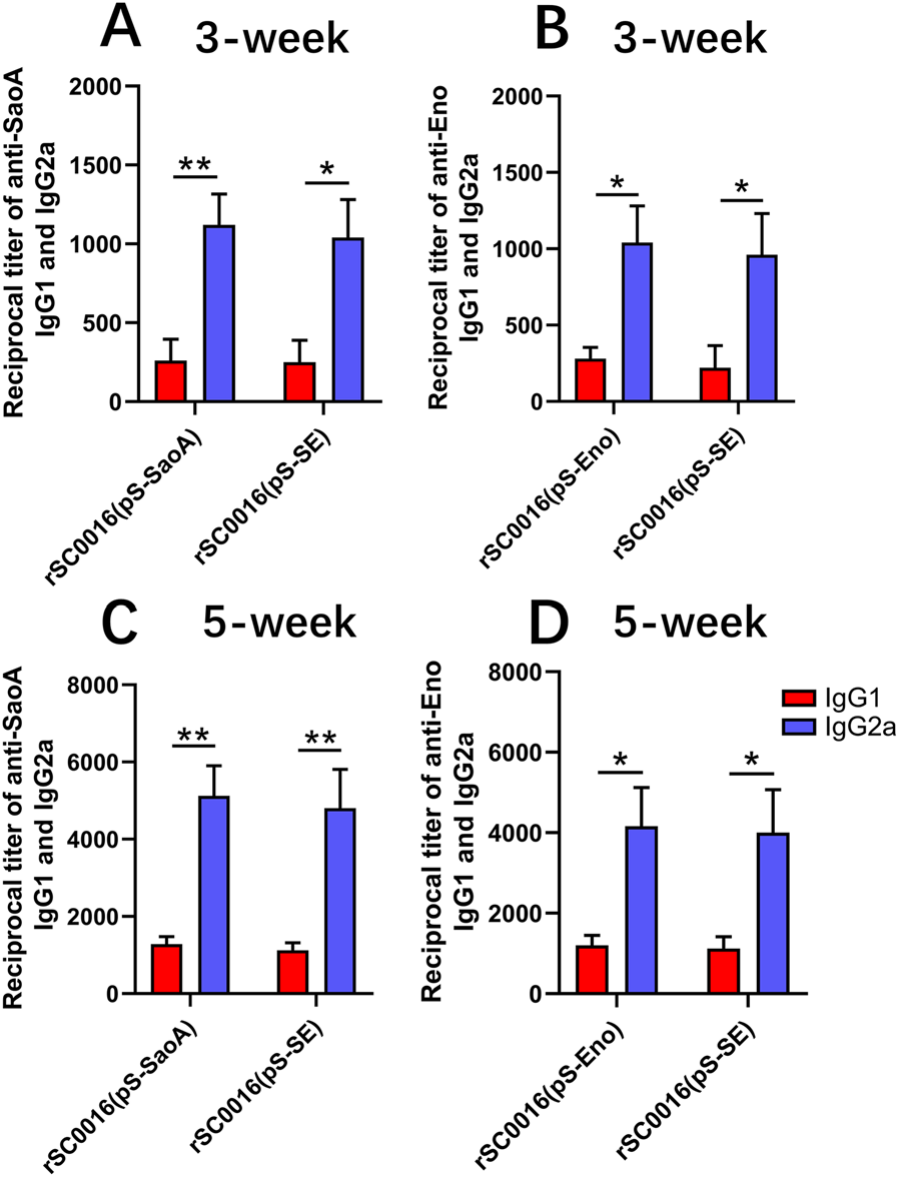
**Serum IgG1 and IgG2a responses to SaoA or Eno.** Serum IgG2a and IgG1 responses to SaoA or Eno were measured by ELISA. The data represent IgG2a and IgG1 subclass antibody titers to SaoA or Eno in sera from ten mice orally immunized with the indicated *S*. Choleraesuis strains 3 weeks after the first immunization and 2 weeks after boosting. Serum from individual mice were serially diluted to obtain titers, starting from 1:50. Error bars represent variation between mice. *ns* not significant. *P < 0.05; **P < 0.01.

The types of immune responses induced by rSC0016(pS-SE) to SaoA or Eno were further evaluated by measuring the levels of IgG isotype IgG1 and IgG2a. The levels of both SaoA-specific and Eno-specific IgG2a were higher than those of IgG1 (Figure 5), suggesting that both rSC0016(pS-SE) carrying a dual expression cassette and rSC0016(pS-SaoA) or rSC0016(pS-Eno) carrying a single expression cassette showed Th1-type dominant immune responses to SaoA or Eno. After booster immunization, there were no significant differences between the Eno-specific IgG1 and IgG2a between rSC0016(pS-SE) and rSC0016(pS-Eno), although rSC0016(pS-SE) induced slightly lower antibody titers than rSC0016(pS-Eno) did (Figure 5D).

### Opsonophagocytic assays

**Figure 6:**
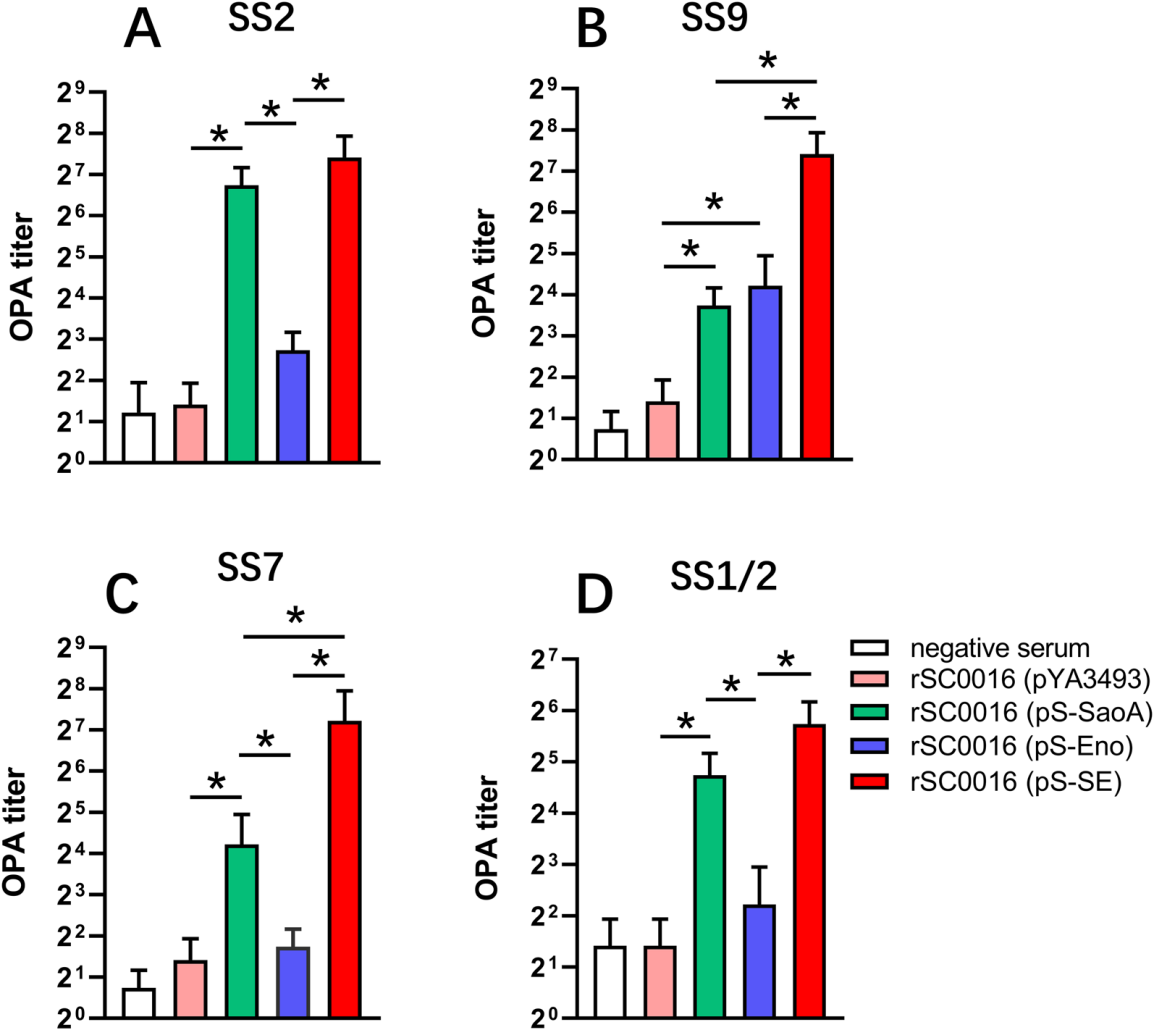
**Bactericidal assays. **Bacterial killing by murine neutrophils under opsonizing conditions. The data represent OPA antibody titers against SS2, SS7, SS9, and SS1/2 in sera from ten mice orally immunized with the indicated ***S***.Choleraesuis strains 2 weeks after boosting. Serum from individual mouse was serially diluted with twofold to obtain titers. Error bars represent variation between mice. SS2, SS7, SS9, and SS1/2 were incubated at 37 ℃ with 5% serum and porcine neutrophils/polymorphonuclear leukocytes (PMNs) at a 1: 1 (CFU: PMN) ratio for 1 h, and bacterial survival was determined as described in the Materials and Methods. *P < 0.05; ***P < 0.001.

The level of opsonizing antibodies, which can be measured by the in vitro OPA, is a better index than total serum antibody titers to reflect the vaccine-induced protection against *S. suis* [3, 35]. To further evaluate the effectiveness of antibodies induced by the rSC0016(pS-SE), the opsonizing activity of mouse serum was measured after booster immunization. The OPA antibody titers induced by rSC0016(pS-SaoA) against SS2, SS9, SS7, and SS1/2 were significantly higher than those of the empty vector rSC0016(pYA3493) (Figures 6A–D). There was no significant difference between rSC0016(pS-Eno) and empty vector rSC0016(pYA3493) although Eno induced a slightly higher titer of OPA antibodies against SS2, SS7 and SS1/2 than that of empty vector. However, the OPA antibody titer induced by rSC0016(pS-Eno) against SS9 was significantly higher than that of empty vector rSC0016(pYA3493) (Figures 6A–D). For homologous strains, the OPA antibody titers against SS2 or SS9 induced by rSC0016(pS-SE) were significantly higher than those of rSC0016(pS-Eno) (Figures 6A and B; *P* < 0.05). In addition, the OPA antibody titer against SS9 induced by rSC0016(pS-SE) was significantly higher than that of rSC0016(pS-SaoA) (Figure 6B; *P* < 0.05). For heterologous strains, the OPA antibody titers against SS7 or SS1/2 of induced by rSC0016(pS-SE) were significantly higher than those of rSC0016(pS-Eno) (Figures 6C and D; *P* < 0.05). In addition, the OPA antibody titer against SS7 induced by rSC0016(pS-SE) was significantly higher than that of rSC0016(pS-SaoA) (Figure 6C; *P* < 0.05). rSC0016(pS-SaoA) is better at inducing a broad OPA reaction against heterologous strains than rSC0016(pS-Eno) which can only induce OPA against homologous strain (Figures 6A–C; *P* < 0.05). In conclusion, a synergetic effect was observed when rSC0016 delivered the dual-antigen expression cassette.

### rSC0016(pS-SE) protects mice against SS2, SS7, SS9 and SS1/2 infection

**Figure 7:**
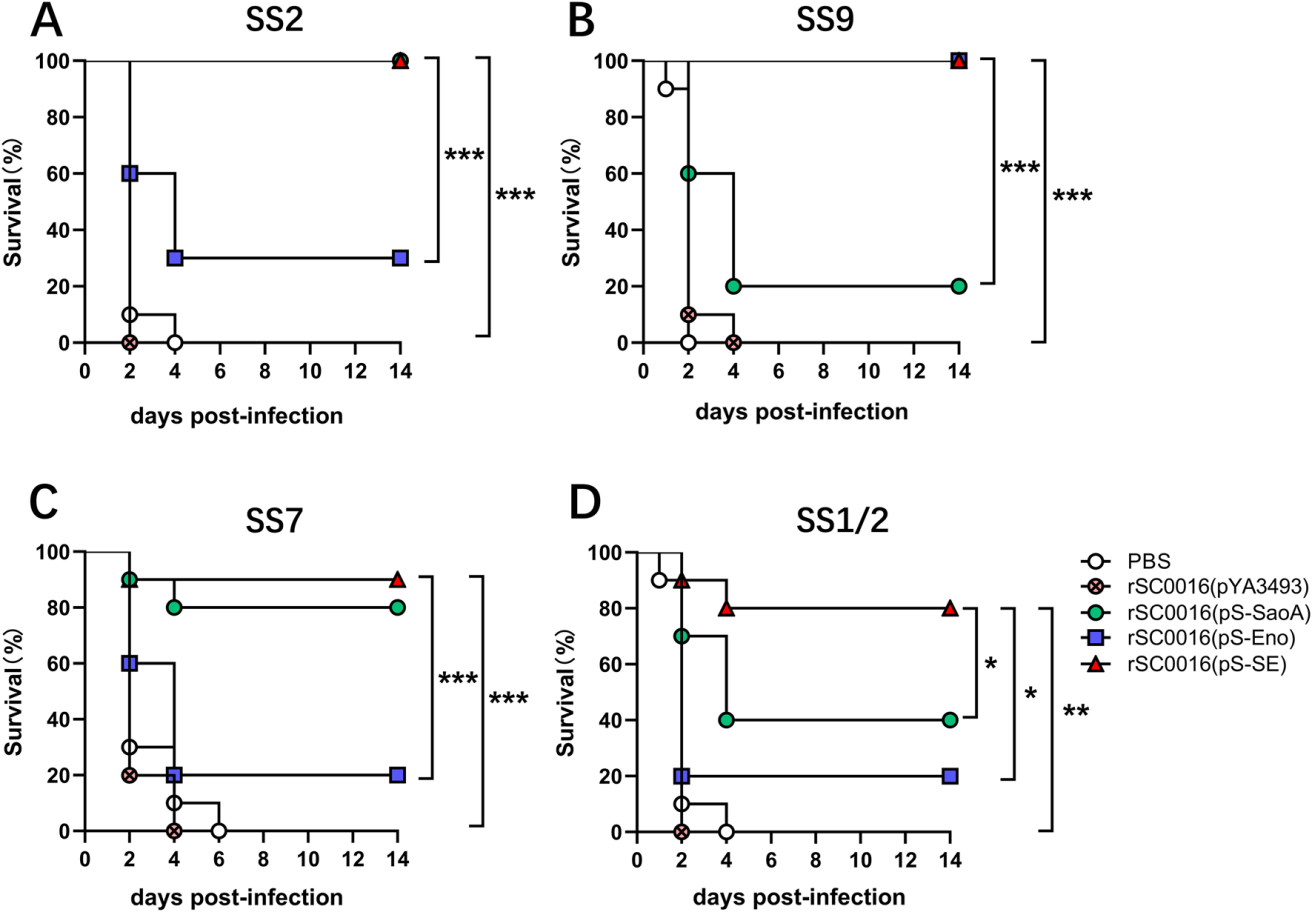
**Survival of immunized mice challenged with wild-type** ***Streptococcus suis*strains SS2, SS7, SS9, or SS1/2.** Female BALB/c mice per group were immunized orally twice at 3-week intervals with 1 × 10 ^9^ CFU of with attenuated *Salmonella* carrying either pS-SaoA, pS-Eno, pS-SE, or pYA3493 (empty vector) and PBS. Each group of mice was randomly divided into 5 subsets of ten mice each and were respectively challenged intraperitoneally with 1.2 × 10^8^ CFU of SS2 (**A**), 1.0 × 10^8^ CFU of SS7 (**B**), 6.3 × 10^8^ CFU of SS9 (**C**), 8.5 × 10^8^ CFU of SS1/2 (**D**) or PBS 2 week after boosting. The challenge dose is equal to 10 × LD_50_. Survival analysis was performed using the Kaplan–Meier method with the log-rank (Mantel-Cox) test. *P < 0.05; **P < 0.01; ***P < 0.001.

**Figure 8:**
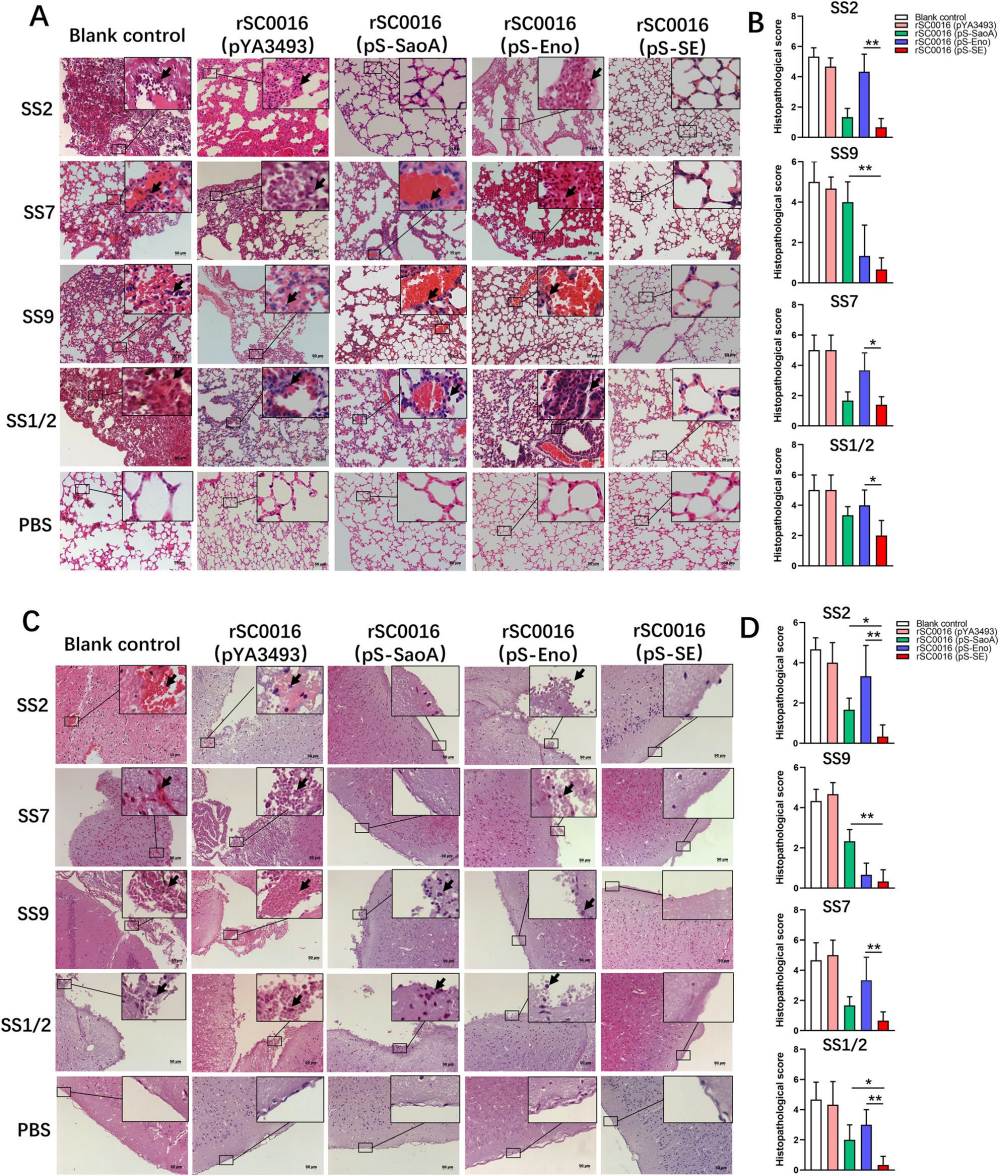
**Histopathology of the lung and brain of the immunized mice infected with** ***S. suis*** **serotypes 2, 7, 9 and ½.** Microscopic pathological observations of lung and brain of mice after challenge with ***S. suis*** serotypes 2, 7, 9 and 1/2. For lung (**A**), neutrophilic infiltration and interstitial thickening are shown with a black arrow. For brain (**C**), meningeal thickening and neutrophilic infiltration are shown with a black arrow. The experiment was performed twice. A representative figure of each group is shown (*n* = 10). The results from both experiments were similar and the data were pooled for analysis. Histology scores in the lung (**B**) and brain (**D**). Horizontal lines show the mean, and error bars represent the SD (*n* = 10). *P < 0.05; **P < 0.01.

To evaluate cross‑protection against multiple serotypes of *S. suis* conferred by rSC0016(pS-SE), immunized mice were challenged with 10 × LD_50_ SS2, SS7, SS9, and SS1/2, respectively. Mice which were given PBS (blank control) or empty vector rSC0016(pYA3493) all died within 6 days after the challenge with different serotypes of *S. suis*. For SS2 or SS7 challenge, the survival rate of mice immunized with rSC0016(pS-SE) was significantly higher than that with rSC0016(pS-Eno) (Figures 7A, C; *P* < 0.001), but similar to rSC0016(pS-SaoA). For SS9 challenge, the survival rate of mice immunized with strain rSC0016(pS-SE) was significantly higher than that with strain rSC0016(pS-SaoA) (Figure 7B; *P* < 0.001), but similar to rSC0016(pS-Eno). For the SS1/2 challenge, the survival rate of mice immunized with strain rSC0016(pS-SE) was significantly higher than that with either strain rSC0016(pS-SaoA) or rSC0016(pS-Eno) (Figure 7D; *P* < 0.05). These results support the view that multiple antigens provide broader protection than a single antigen. In addition, lung tissue section analysis showed severe hyperemia and massive inflammatory exudation in mice immunized with empty vector rSC0016(pYA3493) or PBS, moderate hyperemia and a small amount of inflammatory exudation in mice immunized with rSC0016(pS-Eno) or rSC0016(pS-SaoA), and mild histopathological lesions in mice immunized with rSC0016(pS-SE) (Figures 8A and B). The analysis of brain sections showed that most of the mice were immunized with rSC0016(pYA3493) and a few mice were immunized with rSC0016(pS-Eno) or rSC0016(pS-SaoA) showed meningeal hemorrhage and neutrophil infiltration. The mice immunized with rSC0016(pS-SE) showed mild histopathological lesions (Figures 8C and D). To sum up, rSC0016(pS-SE) presenting dual expression cassette provides stronger and broader protection than rSC0016(pS-SaoA) or rSC0016(pS-Eno) presenting a single antigen.

## Discussion

Vaccine strains with polyvalent antigens need to be developed to cover multiple-serotype of *S. suis* epidemic strains [2, 3, 18]. In this study, rSC0016(pS-SE), which integrates a dual antigen expression cassette, induces antibodies with cross-opsonophagocytic activity and provides high protection against multiple serotypes of *S. suis*.

For *Salmonella* vector, the metabolic pressure or virulence caused by foreign antigens, especially foreign antigens with multiple expression cassettes, needs to be accurately evaluated [33]. On the one hand, it is hard to induce a robust immune response if the expression level of the foreign protein is too low. On the other, it may also cause metabolic pressure on the vector and affect the colonization of the vector in the host lymphatic system if the foreign antigen is overexpressed, which impairs the ability of the vector to induce an effective immune response [34]. Previously, we developed a regulated delayed antigen synthesis and regulated delayed attenuated system of *S.* Choleraesuis, which optimized the colonization and the synthesis of foreign antigens [12, 27–29]. Those studies tested the system with a single antigen. In this study, the system was used to regulate two antigens as an operon fusion. The synthesis of LacI under the arabinose promoter gradually decreased with the decrease of arabinose (Figure 1C). Correspondingly, the production of SaoA and Eno under the P_trc_ promoter regulation gradually increased (Figure 1C). The results demonstrate that both SaoA and Eno were synthesized independently in rSC0016(pS-SE). Both were under the strict control of the delayed synthesis antigen system since the same promoter regulated them. The growth rate and lymphatic tissue distribution of rSC0016(pS-SaoA) are like that of strain rSC0016(pS-SaoA) or rSC0016(pS-Eno), suggesting that the metabolic pressure of the dual expression cassette is not a concern for rSC0016(pS-SE). In summary, rSC0016(pS-SE) with an increased antigen production still achieves a balance between antigen synthesis and colonization, which could be attributed to the presence of the delayed antigen synthesis system.

The antibody titer reflects the degree to which the antigen components in the vaccine are processed and presented. It is usually positively correlated with the antigen concentration contained in the vaccine [36]. In this study, the specific antibody titers (IgG or IgA) against SaoA or Eno induced by rSC0016(pS-SE) strain were significantly higher than those of the empty vector control group (Figures 4A–D) at 3- and 5- weeks after the first immunization, suggesting that rSC0016(pS-SE) strain delivering dual expression cassette could induce SaoA-specific and Eno-specific antibody responses simultaneously. Notably, the level of SaoA-specific antibody (IgG or IgA) induced by rSC0016(pS-SE) in mice is equivalent to that induced by rSC0016(pS-SaoA) (Figures 4A and C). This result demonstrates that the SaoA in rSC0016(pS-SE) retained similar immunogenicity to that in rSC0016(pS-SaoA). In this study, we observed a decrease of Eno-specific antibodies induced by rSC0016(pS-SE) compared to rSC0016(pS-Eno) at 2 weeks after the boost immunization (Figure 4D).

The result may be due to the abundant antigen stimulation brought by the dual expression cassette delivered by rSC0016(pS-SE), which leads to slight immune tolerance in the immune system of mice [37, 38]. Although it is not a significant difference, the production of Eno is slightly lower in rSC0016(pS-SE) than that in pS-Eno. This might also contribute to the lower antibody responses against Eno.

The level of opsonizing antibodies, which can be measured by the in vitro OPA, is one of the indexes to reflect the vaccine-induced protection against *S. suis* [3, 35]. Sao formulated with Emulsigen-Plus® failed to induce OPA antibody against SS2, or SS9 then failed to confer protection but induced OPA antibody against SS2 and conferred protection when formulated with Quil-A [5, 6]. Although antibody-mediated opsonophagocytosis is considered a good indicator of protection against *S. suis* [3, 4, 35], few vaccine candidates exhibit the OPA to heterologous *S. suis* serotype. In this study, rSC0016(pS-SaoA) not only induced OPA antibody against SS2 (Figure 6A), but also induced OPA antibodies to heterologous serotypes SS7, SS9 and SS1/2 (Figures 6B–D), demonstrating that rSC0016 is a suitable vector for SaoA, which is beneficial to enhance the immunogenicity of SaoA. Previous studies have shown that Eno-antiserum from rabbits does not exhibit OPA [39]. However, we observed in this study that rSC0016(pS-Eno) induced mice to produce OPA antibodies against SS9 (Figure 6B). This result indicated that the opsonizing activity of antigen-specific antibodies varied according to adjuvants or delivery systems [6, 40]. Animal species might also affect the OPA induced by the same antigen. Although the protein homology of Eno among these serotypes is high (Table 2), rSC0016(pS-Eno) does not induce cross-OPA antibodies against heterologous serotypes. This result indicated that the cross-reactivity of OPA antibodies might not be positively correlated with antigen conservation. The completion of detailed epitope information will allow a better understanding of the mechanism by which antibodies induce cross-OPA.

**Table 2:** **Amino acid homology of SS2-SaoA or SS9-Eno**.

Homology strains	Coverage (%)
SS2-SaoA VS SS9-SaoA	98.5
SS2-SaoA VS SS7-SaoA	94.2
SS2-SaoA VS SS1/2-SaoA	100
SS9-Eno VS SS2-Eno	99.8
SS9-Eno VS SS7-Eno	99.8
SS9-Eno VS SS1/2-Eno	99.8

It is interesting to note that the antiserum of rSC0016(pS-SE) has a broader and more effective opsonophagocytic response than either rSC0016(pS-SaoA) or rSC0016(pS-Eno) against multiple types of *S. suis* (Figures 6A–D). These results may be due to rSC0016(pS-SE) induced OPA antibodies that can target two antigens. Host protection against highly encapsulated *S. suis* is mainly mediated by opsonophagocytosis, which is closely related to the function of IgG2a [41]. IgG2a, which marks Th1-dominant immune responses, can effectively mediate bacterial lysis through bacterial opsonophagocytosis by triggering the complement cascade [42]. In contrast, IgG1 elicited during Th2-dominant immune responses usually has a less protective potential [41, 42]. In this study, rSC0016 confers a strong Th1-dominant immune response to foreign antigens (Figures 5A–D), which may result in a broad and robust OPA response against multiple serotypes of *S. suis* induced by rSC0016(pS-SE).

In our previous study, high cross-protection against SS7 (80%) and SS9 (100%) was observed in mice when utilizing rSC0016 to deliver SS2-Eno [28]. In this study, rSC0016 delivers SS9-Eno confer only 20% heteroprotection against SS7 challenges.

The type of signal peptide affects the synthesis and secretion of the fusion protein [49]. Antigens secreted to the extracellular of *Salmonella* vector are more readily recognized by antigen-presenting cells to enhance adaptive immunity [30, 49]. Previous studies have shown that the secretory effect of the OmpA signal peptide is weaker than that of the Bla/ss signal peptide [30]. Therefore, we reasonably speculate that the secretory level of SS9-Eno fused with OmpA signal peptide is likely lower than that of SS2-Eno fused with Bla/ss signal peptide, resulting in lower immunogenicity of rSC0016 carrying SS9-Eno than that of rSC0016 carrying SS2-Eno. Interestingly, there is just one amino acid difference at position 250 between the SS2-Eno and the SS9-Eno. A polar amino acid tyrosine is located at position 250 in SS2-Eno, while a non-polar amino acid phenylalanine is at position 250 in SS9-Eno. Position 250 is within the plasminogen binding site [43]. The transition from polar amino acids to non-polar amino acids often leads to a change in protein conformation [44], which could lead to antigen diversity [45]. Our results emphasize the need to test the immunogenicity of homologous antigenic proteins from different serotypes, even if they have very high homology. The protection achieved by rSC0016(pS-SE) against homologous strains SS2 and SS9 atteined 100%, suggesting that dual antigen-containing rSC0016(pS-SE) retained the original immunogenicity of SaoA and Eno. Generally speaking, the more conservative the antigen, the higher the efficiency of heterogeneous protection [9, 12, 21, 28, 46, 47]. SaoA and Eno are highly conserved among the heterologous SS prevalent strains used here (Table 2). Our results showed that the combination of these two conserved antigens improves heterologous protection (Figure 7). It is possible that antigens cooperate with each other to induce an effective immune response [21, 46], or the combination of antigens with different functions provides broader protection [18, 20, 46]. The clinical symptoms of *S. suis* in humans are mainly meningitis [50]. In this study, we observed different degrees of hemorrhage and inflammatory cell infiltration in the brain tissue of mice in the blank control group but not in the rSC0016(pS-SE) immunized group after respectively intraperitoneal injection of SS2, SS9, SS7, and SS9 (Figures 8C, D). This suggests that rSC0016(pS-SE) has a potential against *S. suis*-causing meningitis. These results show that our rSC0016(pS-SE) has the potential to resist *S. suis*-causing meningitis, which provides a new idea for the development of vaccines against *S. suis*-causing meningitis in piglets and humans. Admittedly, mouse trials can only suggest the efficacy of the vaccine to a certain extent. Next step, we will carry out target animal experiments in piglets to further verify the protective efficacy of rSC0016(pS-SE). In conclusion, the recombinant attenuated vector of *S.* Choleraesuis rSC0016 can be used as a factory to produce multivalent antigens from different serotypes of *S. suis* as a vaccine to broaden the cross-protection against multiple serotypes of *S. suis*, which enables *S.* Choleraesuis vector as an effective platform to develop a multivalent vaccine.

## Data Availability

The materials and data not presented in this manuscript are available from the corresponding author upon request.

## Abbreviations

*S*. Choleraesuis: *Salmonella enterica* Serotype choleraesuis
*S. suis*: *Streptococcus suis*
Sao: surface-anchored protein
SS2: *S. suis* Serotype 2
SS7: *S. suis* Serotype 7
SS9: *S. suis* Serotype 9
SS1/2: *S. suis* Serotype ½
LB: Luria broth
LD_50_: 50% Lethal dose
Th1: T helper type 1
Th2: T helper type 2
PBS: phosphate buffer saline
SDS-PAGE: sodium dodecyl sulfate–polyacrylamide
MRP: muramidase-released protein
EF: extracellular factor
FCA: Freund’s complete adjuvant
OPA: opsonophagocytic activity

## References

[1] Gottschalk M, Xu J, Calzas C, Segura M (2010). *Streptococcus suis*: a new emerging or an old neglected zoonotic pathogen?. Future Microbiol.

[2] Goyette-Desjardins G, Auger JP, Xu J, Segura M, Gottschalk M (2014). *Streptococcus suis*, an important pig pathogen and emerging zoonotic agent-an update on the worldwide distribution based on serotyping and sequence typing. Emerg Microbes Infect.

[3] Segura M (2015). *Streptococcus suis* vaccines: candidate antigens and progress. Expert Rev Vaccines.

[4] Kock C, Beineke A, Seitz M, Ganter M, Waldmann KH, Valentin-Weigand P, Baums CG (2009). Intranasal immunization with a live *Streptococcus suis* isogenic ofs mutant elicited suilysin- neutralization titers but failed to induce opsonizing antibodies and protection. Vet Immunol Immunopathol.

[5] Baums CG, Kock C, Beineke A, Bennecke K, Goethe R, Schroder C, Waldmann KH, Valentin- Weigand P (2009). *Streptococcus suis* bacterin and subunit vaccine immunogenicities and protective efficacies against serotypes 2 and 9. Clin Vaccine Immunol.

[6] Li Y, Gottschalk M, Esgleas M, Lacouture S, Dubreuil JD, Willson P, Harel J (2007). Immunization with recombinant Sao protein confers protection against *Streptococcus suis* infection. Clin Vaccine Immunol.

[7] Dekker CN, Bouma A, Daemen AJ, van Leengoed LA, Jonker FH, Wagenaar JA, Stegeman JA (2012). Homologous whole bacterin vaccination is not able to reduce *Streptococcus suis* serotype 9 strain 7997 transmission among pigs or colonization. Vaccine.

[8] Buttner N, Beineke A, de Buhr N, Lilienthal S, Merkel J, Waldmann KH, Valentin-Weigand P, Baums CG (2012). Streptococcus suis serotype 9 bacterin immunogenicity and protective efficacy. Vet Immunol Immunopathol.

[9] Weisse C, Dittmar D, Jakobczak B, Florian V, Schutze N, Alber G, Klose K, Michalik S, Valentin- Weigand P, Volker U, Baums CG (2021). Immunogenicity and protective efficacy of a *Streptococcus suis* vaccine composed of six conserved immunogens. Vet Res.

[10] Lee SE, Kim SY, Jeong BC, Kim YR, Bae SJ, Ahn OS, Lee JJ, Song HC, Kim JM, Choy HE, Chung SS, Kweon MN, Rhee JH (2006). A bacterial flagellin, Vibrio vulnificus FlaB, has a strong mucosal adjuvant activity to induce protective immunity. Infect Immun.

[11] Hsueh KJ, Lee JW, Hou SM, Chen HS, Chang TC, Chu CY (2014). Evaluation on a *Streptococcus suis* vaccine using recombinant sao-l protein manufactured by bioreactors as the antigen in pigs. Transbound Emerg Dis.

[12] Li Y, Ji Z, Wang X, Wang S, Shi H (2017). *Salmonella enterica* serovar Choleraesuis vector delivering SaoA antigen confers protection against *Streptococcus suis* serotypes 2 and 7 in mice and pigs. Vet Res.

[13] Zhang A, Chen B, Mu X, Li R, Zheng P, Zhao Y, Chen H, Jin M (2009). Identification and characterization of a novel protective antigen, Enolase of *Streptococcus suis* serotype 2. Vaccine.

[14] Jiang X, Yang Y, Zhou J, Liu H, Liao X, Luo J, Li X, Fang W (2019). Peptidyl isomerase PrsA is surface-associated on *Streptococcus suis* and offers cross-protection against serotype 9 strain. FEMS Microbiol Lett.

[15] Rivera-Hernandez T, Rhyme MS, Cork AJ, Jones S, Segui-Perez C, Lawrenz M, Goldblatt D, Collin N, Walker MJ, Kaufmann SHE (2020). Vaccine-induced Th1-type response protects against invasive group A *Streptococcus* infection in the absence of opsonizing antibodies. Mbio.

[16] Esgleas M, Li Y, Hancock MA, Harel J, Dubreuil JD, Gottschalk M (2008). Isolation and characterization of α-enolase, a novel fibronectin-binding protein from *Streptococcus suis*. Microbiology.

[17] Olin P, Rasmussen F, Gustafsson L, Hallander HO, Heijbel H (1997). Randomised controlled trial of two-component, three-component, and five-component acellular pertussis vaccines compared with whole-cell pertussis vaccine. Ad Hoc group for the study of pertussis vaccines. Lancet.

[18] Zhang S, Sella M, Sianturi J, Priegue P, Shen D, Seeberger PH (2021). Discovery of oligosaccharide antigens for semi-synthetic glycoconjugate vaccine leads against *Streptococcus suis* serotypes 2, 3, 9 and 14*. Angew Chem Int Ed Engl.

[19] Wisselink HJ, Vecht U, Stockhofe-Zurwieden N, Smith HE (2001). Protection of pigs against challenge with virulent *Streptococcus suis* serotype 2 strains by a muramidase-released protein and extracellular factor vaccine. Vet Rec.

[20] Marin-Lopez A, Otero-Romero I, de la Poza F, Menaya-Vargas R, Calvo-Pinilla E, Benavente J, Martinez-Costas JM, Ortego J (2014). VP2, VP7, and NS1 proteins of bluetongue virus targeted in avian reovirus muNS-Mi microspheres elicit a protective immune response in IFNAR(-/-) mice. Antiviral Res.

[21] Giuliani MM, Adu-Bobie J, Comanducci M, Arico B, Savino S, Santini L, Brunelli B, Bambini S, Biolchi A, Capecchi B, Cartocci E, Ciucchi L, Di Marcello F, Ferlicca F, Galli B, Luzzi E, Masignani V, Serruto D, Veggi D, Contorni M, Morandi M, Bartalesi A, Cinotti V, Mannucci D, Titta F, Ovidi E, Welsch JA, Granoff D, Rappuoli R, Pizza M (2006). A universal vaccine for serogroup B meningococcus. Proc Natl Acad Sci USA.

[22] Galen JE, Curtiss R (2014). The delicate balance in genetically engineering live vaccines. Vaccine.

[23] Loh FK, Nathan S, Chow SC, Fang CM (2021). Immunogenicity and protection efficacy of enhanced fitness recombinant *Salmonella* Typhi monovalent and bivalent vaccine strains against acute toxoplasmosis. Pathog Glob Health.

[24] Su H, Liu Q, Bian X, Wang S, Curtiss R, Kong Q (2021). Synthesis and delivery of *Streptococcus pneumoniae* capsular polysaccharides by recombinant attenuated *Salmonella* vaccines. Proc Natl Acad Sci USA.

[25] Pfister SP, Schären OP, Beldi L, Printz A, Notter MD, Mukherjee M, Li H, Limenitakis JP, Werren JP, Tandon D, Cuenca M, Hagemann S, Uster SS, Terrazos MA, Gomez De Agüero M, Schürch CM, Coelho FM, Curtiss R, Slack E, Balmer ML, Hapfelmeier S (2020). Uncoupling of invasive bacterial mucosal immunogenicity from pathogenicity. Nat Commun.

[26] Mata-Haro V, Cekic C, Martin M, Chilton PM, Casella CR, Mitchell TC (2007). The vaccine adjuvant monophosphoryl lipid A as a TRIF-biased agonist of TLR4. Science.

[27] Ji Z, Shang J, Li Y, Wang S, Shi H (2015). Live attenuated *Salmonella enterica* serovar Choleraesuis vaccine vector displaying regulated delayed attenuation and regulated delayed antigen synthesis to confer protection against *Streptococcus suis* in mice. Vaccine.

[28] Li Q, Lv Y, Li YA, Du Y, Guo W, Chu D, Wang X, Wang S, Shi H (2020). Live attenuated *Salmonella enterica* serovar Choleraesuis vector delivering a conserved surface protein enolase induces high and broad protection against *Streptococcus suis* serotypes 2, 7, and 9 in mice. Vaccine.

[29] Li Y, Chen Y, Du YZ, Guo W, Chu D, Fan J, Wang X, Bellefleur M, Wang S, Shi H (2020). Live- attenuated *Salmonella enterica* serotype Choleraesuis vaccine with regulated delayed fur mutation confer protection against *Streptococcus suis* in mice. BMC Vet Res.

[30] Xin W, Wanda S, Li Y, Wang S, Mo H, Curtiss R (2008). Analysis of type II secretion of recombinant pneumococcal PspA and PspC in a *Salmonella enterica* Serovar Typhimurium vaccine with regulated delayed antigen synthesis. Infect Immun.

[31] Zhang A, Chen B, Li R, Mu X, Han L, Zhou H, Chen H, Meilin J (2009). Identification of a surface protective antigen, HP0197 of *Streptococcus suis* serotype 2. Vaccine.

[32] Domingues E, Brillet T, Vasseur C, Agier V, Marden MC, Baudin-Creuza V (2009). Construction of a new polycistronic vector for over-expression and rapid purification of human hemoglobin. Plasmid.

[33] Wang S, Li Y, Shi H, Sun W, Roland KL, Curtiss R (2011). Comparison of a regulated delayed antigen synthesis system with in vivo-inducible promoters for antigen delivery by live attenuated *Salmonella* vaccines. Infect Immun.

[34] Li Y, Wang S, Scarpellini G, Gunn B, Xin W, Wanda S, Roland KL, Curtiss R (2009). Evaluation of new generation *Salmonella enterica* serovar Typhimurium vaccines with regulated delayed attenuation to induce immune responses against PspA. Proc Natl Acad Sci USA.

[35] Baums CG, Kock C, Beineke A, Bennecke K, Goethe R, Schroder C, Waldmann KH, Valentin-Weigand P (2009). *Streptococcus suis* bacterin and subunit vaccine immunogenicities and protective efficacies against serotypes 2 and 9. Clin Vaccine Immunol.

[36] DiazGranados CA, Dunning AJ, Robertson CA, Talbot HK, Landolfi V, Greenberg DP (2015). Efficacy and immunogenicity of high-dose influenza vaccine in older adults by age, comorbidities, and frailty. Vaccine.

[37] Walton KL, Galanko JA, Balfour SR, Fisher NC (2006). T cell-mediated oral tolerance is intact in germ-free mice. Clin Exp Immunol.

[38] Birmingham JM, Patil S, Li XM, Busse PJ (2013). The effect of oral tolerance on the allergic airway response in younger and aged mice. J Asthma.

[39] Esgleas M, Dominguez-Punaro ML, Li Y, Harel J, Dubreuil JD, Gottschalk M (2009). Immunization with SsEno fails to protect mice against challenge with *Streptococcus suis* serotype 2. FEMS Microbiol Lett.

[40] Li Y, Martinez G, Gottschalk M, Lacouture S, Willson P, Dubreuil JD, Jacques M, Harel J (2006). Identification of a surface protein of *Streptococcus suis* and evaluation of its immunogenic and protective capacity in pigs. Infect Immun.

[41] Charland N, Jacques M, Lacouture S, Gottschalk M (1997). Characterization and protective activity of a monoclonal antibody against a capsular epitope shared by *Streptococcus suis* serotypes 1, 2 and 1/2. Microbiology.

[42] Chabot-Roy G, Willson P, Segura M, Lacouture S, Gottschalk M (2006). Phagocytosis and killing of *Streptococcus suis* by porcine neutrophils. Microb Pathog.

[43] Lu Q, Lu H, Qi J, Lu G, Gao GF (2012). An octamer of enolase from *Streptococcus suis*. Protein Cell.

[44] Eriksen MB, Nielsen MF, Brusgaard K, Tan Q, Andersen MS, Glintborg D, Gaster M (2013). Genetic alterations within the DENND1A gene in patients with polycystic ovary syndrome (PCOS). PLoS ONE.

[45] Pellequer JL, Westhof E, Van Regenmortel MH (1991). Predicting location of continuous epitopes in proteins from their primary structures. Methods Enzymol.

[46] Darrieux M, Miyaji EN, Ferreira DM, Lopes LM, Lopes AP, Ren B, Briles DE, Hollingshead SK, Leite LC (2007). Fusion proteins containing family 1 and family 2 PspA fragments elicit protection against *Streptococcus pneumoniae* that correlates with antibody-mediated enhancement of complement deposition. Infect Immun.

[47] Ridda I, Musher DM (2012). Is there a potential role for protein-conjugate pneumococcal vaccine in older adults?. Australas Med J.

[48] Roland K, Curtiss R, Sizemore D (1999). Construction and evaluation of a delta cya delta crp *Salmonella* typhimurium strain expressing avian pathogenic *Escherichia coli* O78 LPS as a vaccine to prevent airsacculitis in chickens. Avian Dis.

[49] Kang HY, Srinivasan J, Curtiss R (2002). Immune responses to recombinant pneumococcal PspA antigen delivered by live attenuated *Salmonella enterica* serovar typhimurium vaccine. Infect Immun.

[50] Susilawathi NM, Tarini N, Fatmawati N, Mayura P, Suryapraba A, Subrata M, Sudewi A, Mahardika GN (2019). *Streptococcus suis*-associated meningitis, Bali, Indonesia, 2014–2017. Emerg Infect Dis.

